# Supplementary material for: Extracellular Vesicles Profiling in Acute Myeloid Leukemia Cell Lines: A Proteomic Characterization
Source: Cells. 2025 Oct 22;14(21):1651. doi: 10.3390/cells14211651 (PMC12608589; doi:10.3390/cells14211651)
Supplement: Supplementary file 1 [file cells-14-01651-s001.zip › cells-3773631-supplementary.pdf]

Supplementary materials

Supplementary Table S1. Protein identification: List of the identified proteins in the AML-EVs. Table reports the raw data, the protein quantification and the main downstream and upstream regulators obtained through Ingenuity Pathways Analysis (IPA) for each under investigated conditions. These last sheets report the p-value of overlap and the target molecules of the dataset for each downstream or upstream.

Supplementary Table S2. EVs average count rate and protein concentration.

Sample	Count rate (kcps)	Protein (µg/µl)
OCI-AML-2	227.4	0.3
OCI-AML-3	230.8	0.4
HL-60	227.4	0.4

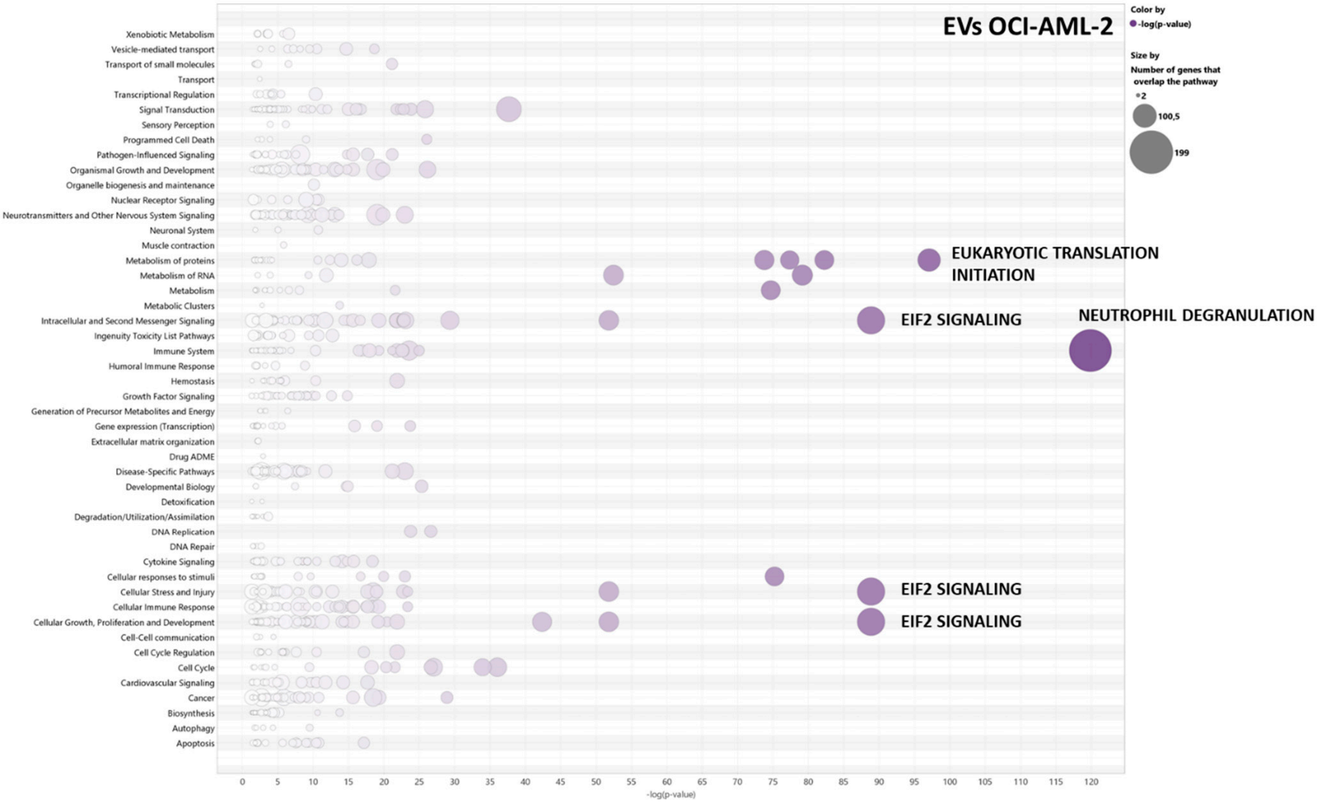

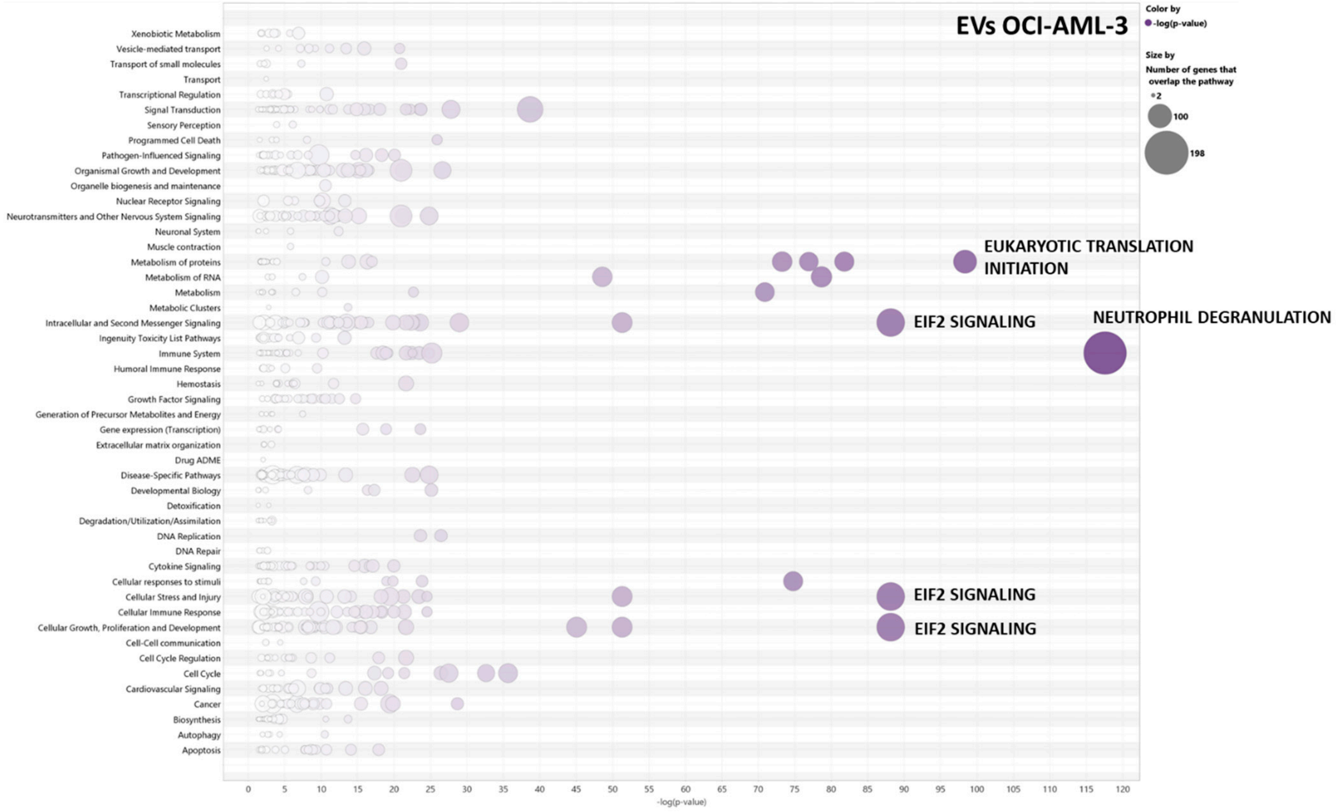

Figure S1. Overview of expression analysis as Canonical Pathways for OCI-AML-2 and OCI-AML-3 EVs. *"Neutrophil Degranulation"* (-Log(p-value) = 120 and 118 for OCI-AML-2 and OCI-AML-3 EVs, respectively), *"Eukaryotic Translation Initiation"* (-Log(p-value) = 97.1 and 98.4 for OCI-AML-2 and OCI-AML-3 EVs, respectively), and *"EIF2 Signaling"* (-Log(p-value) = 88.9 and 88.2, for OCI-AML-2 and OCI-AML-3 EVs, respectively) .

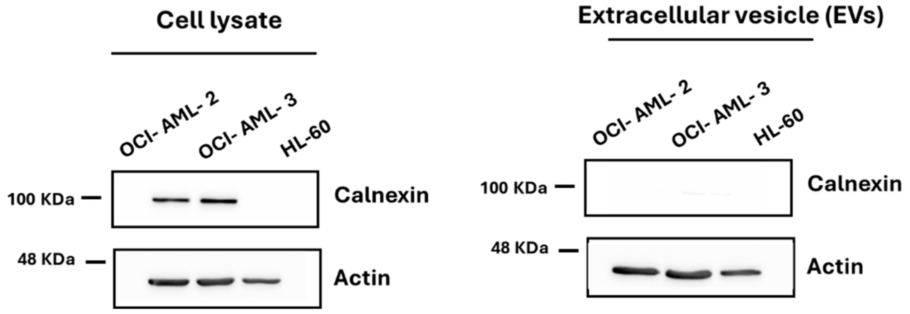

Figure S2. Characterization of EVs isolated from acute myeloid leukemia (AML). Western blot analysis of negative EVs marker Calnexin and actin in leukemia whole cell lysates (A) and in EV lysates (B).

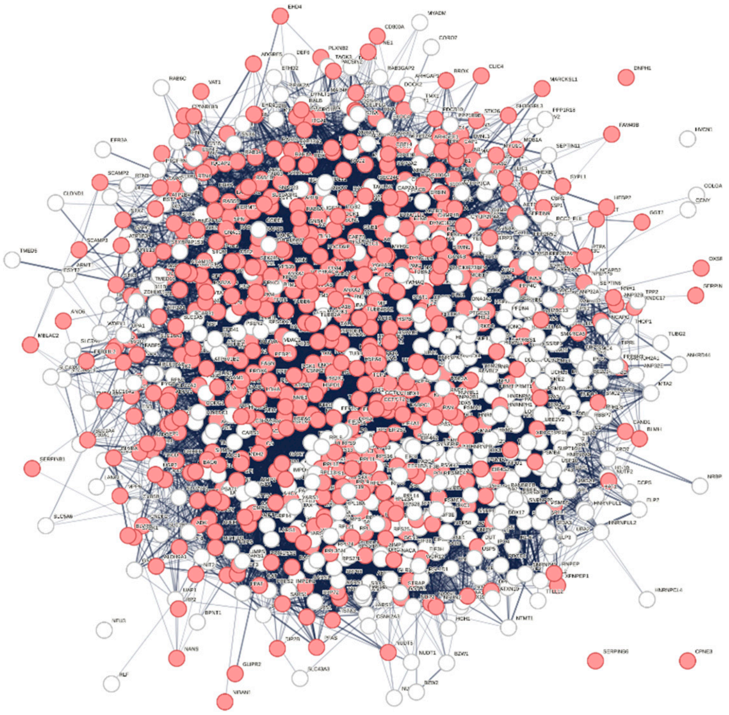

Figure S3. PANTHER Molecular Function Reclassification analysis of EV proteins unmatched with Vesiclepedia repository database. PPI network analysis (PPI enrichment p-value < 1.0 × 10^{-16}) of the quantified common proteins in EVs isolated from HL-60, OCI-AML-2 and OCI-AML-3 culture media. 535 of the 1004 common EV proteins are involved in "Extracellular Exosome" (GO:0070062) as reported by red dots
